# Assessment of dietary magnesium intake in the Eastern Province of Saudi Arabia

**DOI:** 10.25122/jml-2023-0279

**Published:** 2023-12

**Authors:** Ali Mohsen Abualrahi, Fatimah Habib Alhanabi, Rahaf Saeed Alalloush, Zainab Hashim Alsalman, Waleed Ibrahim Albaker, Mona Hmoud AlSheikh, Fatimah Abdulwahed Almuzain, Mohammed Taha Al-Hariri

**Affiliations:** 1 College of Medicine, Imam Abdulrahman Bin Faisal University, Dammam, Saudi Arabia; 2 Eastern Health Cluster, Dammam, Saudi Arabia; 3 Department of Internal Medicine, College of Medicine, Imam Abdulrahman Bin Faisal University, Dammam, Saudi Arabia; 4 Department of Physiology, College of Medicine, Imam Abdulrahman Bin Faisal University, Dammam, Saudi Arabia; 5 Diabetes Unit, King Fahd Hospital, Riyadh, Saudi Arabia

**Keywords:** magnesium, intake, Saudi, questionnaire

## Abstract

Magnesium is an essential element and the most abundant intracellular cation after potassium. This cross-sectional study assessed the average dietary magnesium intake among residents of the Eastern Province of Saudi Arabia. Data was collected using a self-reported validated electronic questionnaire between April 2022 and July 2023. The first part of the survey included demographic data, and the second section comprised 33 items, including a semi-quantitative tool specifically designed to evaluate magnesium intake over the last three months. We included 1065 participants, out of whom 61.1% were women. The predominant age group was 19 – 26 years (56.9%), and most participants (83.3%) reported no comorbidities. The majority (48.5%) had normal weight, 246 (23%) were overweight, and 193 (18.1%) were obese. Most participants had low dietary magnesium intake, ranging from one to five times monthly. There was a positive correlation between age and dietary magnesium consumption. The study highlights a concerning trend of low magnesium intake, representing a risk for various chronic diseases. This trend could be linked to increased consumption of typical diets low in magnesium, such as those high in refined sugars, flour, and processed foods, prevalent among the younger Saudi population.

## INTRODUCTION

Magnesium is an essential element and the most abundant intracellular cation after potassium. It plays a vital role in regulating the human body by acting as an enzyme cofactor in multiple metabolic reactions involving the metabolism of carbohydrates, proteins, and fats, nerve conduction, energy production, and blood pressure [1]. In addition, magnesium is an activator for all thiamine pyrophosphate reactions, and it is an important cofactor for phosphate transfer reactions and cellular respiration involving adenosine mono-, di-, and triphosphate. Some of the processes in which magnesium is a cofactor include but are not limited to, stabilizing mitochondrial membranes and DNA and RNA synthesis [2].

Prior evidence indicated that low magnesium intake was inversely associated with insulin resistance [3] and dyslipidemia [4] and their outcomes, including type 2 diabetes [3], hypertension [5], cardiovascular disease [6], and cancer [7]. Hypomagnesemia promotes an inflammatory response, ultimately leading to several inflammatory diseases, such as metabolic disease and endothelial dysfunction [4].

According to the United States Food and Nutrition Board, the daily recommended requirements to meet the daily allowance for adult males and females are 420 mg and 320 mg, respectively [8]. Interestingly, magnesium supplementation reduces insulin resistance and improves long-term glycemic control indicators in patients with type 2 diabetes mellitus [9].

Food intake surveys are cost-effective and convenient tools commonly employed in clinical research to assess nutrient consumption [10]. The consumption of magnesium-rich foods, such as green vegetables, seeds, grains, and nuts, aids in fundamental processes [1].

Hypomagnesemia is defined as a serum magnesium level below 1.6 mg/dL (0.66 mmol/L), with clinically significant symptoms and signs typically manifesting when serum magnesium concentration falls below 1.2 mg/dL (0.5 mmol/L) [11]. Common causes of hypomagnesemia include severely low dietary intake, gastrointestinal malabsorptive states, acute pancreatitis, and renal hyperfiltration associated with diabetes mellitus, as well as medications such as proton pump inhibitors, calcineurin inhibitors, antibiotics (e.g., aminoglycosides, amphotericin B), cytotoxic drugs, and diuretics [12, 13].

On the other hand, hypomagnesemia can lead to several clinical conditions, including insulin resistance, cardiovascular disease, central obesity, and asthma. Studies have shown that hypomagnesemia can result from genetic or acquired causes. The acquired causes can be decreased intake, impaired gastrointestinal (GI) absorption, increased renal losses, or drugs [12]. The clinical signs and symptoms of hypomagnesemia are non-specific, but symptoms start to appear when serum magnesium levels fall below 0.5 mmol/L (1.2 mg/dL) [12]. Hypomagnesemia has a clinical effect on the neuromuscular, renal, cardiovascular, and gastrointestinal systems, increasing the risk of developing severe chronic illnesses [1].

Despite magnesium-rich foods in the Saudi diet, including dried fruits, dairy products, seeds, and nuts [14], studies on the prevalence of magnesium consumption in Saudi Arabia are limited and mainly related to other comorbidities [15]. Therefore, this study aimed to estimate the average magnesium intake in the Eastern Province of Saudi Arabia. This assessment is vital as it could assist in the early detection of hypomagnesemia and its related health risks in clinical practice and generate recommendations that could enhance research focused on magnesium and its health implications.

## MATERIAL AND METHODS

This cross-sectional study was conducted from April 2022 to July 2023 at King Fahd Hospital of the University and Imam Abdulrahman bin Faisal University (IAU), including the surrounding areas in the Eastern Province of Saudi Arabia. Before participation, all individuals were thoroughly screened for eligibility. We enrolled Saudi participants aged 18 to 65 years with no history of medications that might affect magnesium intake or absorption, such as proton pump inhibitors, calcineurin inhibitors, antibiotics (e.g., aminoglycosides, amphotericin B), cytotoxic drugs, and diuretics [13]. Geriatric participants more than 65 years of age were excluded from the study.

### Data collection

Data was collected using a self-reported validated electronic questionnaire, ensuring participant confidentiality. The questionnaire, designed to assess magnesium food intake, was divided into two main sections. The first section collected demographic information, including age, gender, education level, comorbidities, smoking and vitamin consumption, and body mass index (BMI) [16]. The second section included 33 items forming a semi-quantitative tool to capture magnesium intake over the last three months. This was a valuable method to determine magnesium consumption, focusing on foods rich in magnesium (>5% daily value). The survey included a variety of magnesium-rich foods, such as dairy products (milk, yogurt), seeds and nuts (sunflower, pumpkin, cashews, or pecans seeds), fish (mackerel, salmon), grains (white bread, whole wheat bread, brown rice) and dark leafy greens (collards, spinach, kale). The magnesium content in these foods ranged from 6 mg per serving in white bread to 178 mg per serving in cashews [17].

The translation of the questionnaire adhered to the World Health Organization (WHO) guidelines, employing a forward/backward (multi-step) procedure. Initially, the questionnaire was translated from English to Arabic by Saudi specialists, including consultants in internal medicine and medical education who were proficient in English, who translated the list of items into Arabic. Subsequently, a back translation was implemented, and the Arabic version was translated back into English. This step was crucial to ensure the consistency and accuracy of the translation. The back-translated version was then reviewed by a bilingual expert with expertise in medical and clinical nutrition. This process produced a single, reliable, translated questionnaire version [18].

### Statistical analysis

Data was assessed and analyzed using IBM SPSS.22. Spearman’s rank correlation coefficient analysis was used to determine the relationship between age, gender, education status, comorbidities, BMI, and daily dietary magnesium intake. A significance level (P value) of less than 0.05 was set for statistical significance.

## RESULTS

The current study included 1,065 participants, out of whom 651 (61.1%) were women and 414 (38.9%) were men, ranging in age from 15 to 65 years. Most participants (606, 56.9%) were between 19 and 26 years old. Of 1,065 participants, 707 (66.4%) were graduates, 266 (25%) had high school certificates, and 1 had no formal education. The majority (887, 83.3%) did not have any comorbidity, 50 (4.7%) had asthma, 36 (3.4%) were hypertensive, and 42 (3.9%) had diabetes. Other characteristics of the study population are presented in Table 1.

**Table 1:** Participant demographics (n = 1065)

		Frequency	Percent
**Age** Years	≤ 18	125	11.7
	19-29	606	56.9
	30-39	125	11.7
	40-49	143	13.4
	50-65	66	6.2
**Gender**	Male	414	38.9
	Female	651	61.1
**Education**	No formal education	1	0.1
	Secondary or less	38	3.6
	High school certificate	266	25
	Graduate	707	66.4
	Postgraduate	53	5
**Comorbidities**	T1D	25	2.3
	T2DM	17	1.6
	Hypertension	36	3.4
	Obesity	33	3.1
	Asthma	50	4.7
	Thyroid disorder	16	1.5
	Celiac disease	1	0.1
	None	887	83.3
**Smoking**	Smoker	115	10.8
	Non-smoker	831	78
	Exposed to secondhand smoke	119	11.2
**Vitamins**	Vitamin D	161	15.1
	Calcium	35	3.3
	Magnesium	17	1.6
	Multi-vitamin and mineral	196	18.4
	No nutritional supplements	656	61.6

T1D: type 1 diabetes mellitus; T2DM: type 2 diabetes mellitus

**Figure 1:**
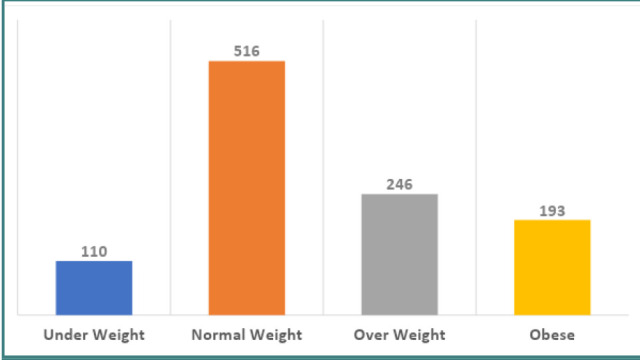
Body mass index (n = 1,065)

**Figure 2:**
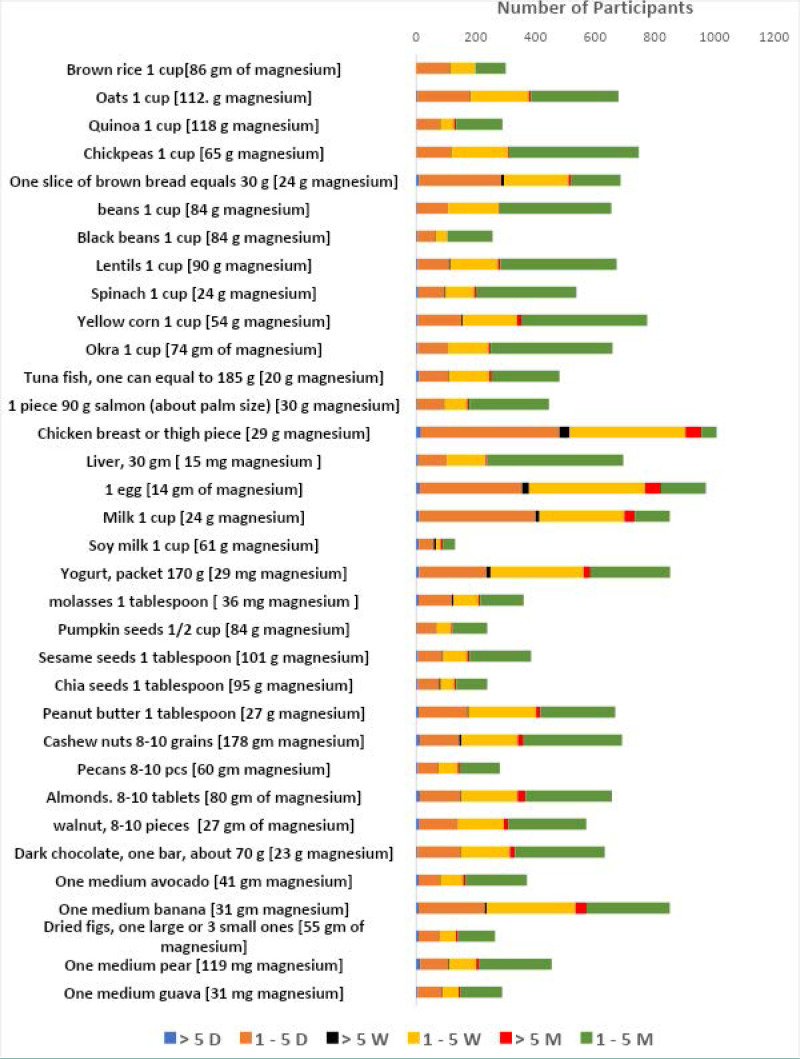
Monthly dietary magnesium intake among participants (n = 1,065)

Of the 1,065 participants interviewed, 516 (48.5%) had normal weight, 246 (23%) were overweight, and 193 (18.1%) were obese (Figure 1). The frequency of dietary magnesium intake among participants is presented in Figure 2. It was observed that the majority had a low frequency of magnesium consumption, typically one to five times per month. The relationship between dietary magnesium consumption and age, gender, and BMI is presented in Table 2. Daily dietary magnesium intake increased with increasing age (P < 0.05).

**Table 2:** Correlation between daily dietary magnesium consumption and age, gender, and BMI

	Age	Sex Ŧ	BMI
Brown rice 1 cup [86 gm of magnesium]	0.009	-0.064*	-0.017
Oats 1 cup [112 g magnesium]	0.122*	0.139*	-0.004
Quinoa 1 cup [118 g magnesium]	0.069*	0.080*	-0.032
Chickpeas 1 cup [65 g magnesium]	0.130*	-0.124*	0.049
One slice of brown bread equals 30 g [24 g magnesium]	0.155*	0.007	0.144*
beans 1 cup [84 g magnesium]	0.217*	-0.095*	0.116*
Black beans 1 cup [84 g magnesium]	0.036	-0.027	-0.018
Lentils 1 cup [90 g magnesium]	0.152*	0.044	0.047
Spinach 1 cup [24 g magnesium]	0.110*	0.083*	0.068*
Yellow corn 1 cup [54 g magnesium]	-0.007	0.113*	-0.049
Okra 1 cup [74 gm of magnesium]	0.211*	0.007	0.089*
Tuna fish, one can equal to 185 g [20 g magnesium]	0.024	-0.03	0.110*
1 piece 90 g salmon (about palm size) [30 g magnesium]	0.061*	-0.048	0.01
Chicken breast or thigh piece [29 g magnesium]	0.021	-0.013	0.052
Liver, 30 gm [ 15 mg magnesium]	0.195*	-0.177*	0.145*
1 egg [14 gm of magnesium]	0.142*	-0.059	0.147*
Milk 1 cup [24 g magnesium]	-0.041	-0.059	0.012
Soy milk 1 cup [61 g magnesium]	0.014	0.005	-0.047
Yogurt, packet 170 g [29 mg magnesium]	0.120*	0.049	0.043
molasses 1 tablespoon [ 36 mg magnesium]	0.029	0.039	-0.015
Pumpkin seeds 1/2 cup [84 g magnesium]	0.173*	0.051	0.088*
Sesame seeds 1 tablespoon [101 g magnesium]	0.077*	0.011	0.037
Chia seeds 1 tablespoon [95 g magnesium]	0.110*	0.095*	-0.013
Peanut butter 1 tablespoon [27 g magnesium]	0.152*	-0.076*	0.106*
Cashew nuts 8-10 grains [178 gm magnesium]	0.149*	0.001	0.063*
Pecans 8-10 pcs [60 gm magnesium]	0.141*	0.096*	0.024
Almonds. 8-10 tablets [80 gm of magnesium]	0.185*	0.039	0.048
walnut, 8-10 pieces [27 gm of magnesium]	0.196*	0.039	0.051
Dark chocolate, one bar, about 70 g [23 g magnesium]	0.093*	-0.004	0.008
One medium avocado [41 gm magnesium]	0.107*	0.047	0.031
One medium banana [31 gm magnesium]	0.149*	-0.088*	0.110*
Dried figs, one large or 3 small ones [55 gm of magnesium]	0.289*	0.03	0.097*
One medium pear [119 mg magnesium]	0.170*	0.081*	0.042
One medium guava [31 mg magnesium]	0.164*	0.014	0.048

* Significant correlation (P values < 0.05), Ŧ Positive correlation related to female participants and negative correlation related to male participants

Consumption of dietary magnesium sources like oats (1 cup with 112 g magnesium), quinoa (1 cup with 118 g magnesium), lentils (1 cup with 90 g magnesium), spinach (1 cup with 24 g magnesium), chia seeds (1 tablespoon with 95 g magnesium), pecans (8-10 pcs with 60 g magnesium), and one medium pear (119 mg magnesium) was significantly correlated with female participants. On the other hand, foods such as brown rice (1 cup with 86 g magnesium), chickpeas (1 cup with 65 g magnesium), beans (1 cup with 84 g magnesium), black beans (1 cup with 84 g magnesium), liver (30 g with 15 mg magnesium), peanut butter (1 tablespoon with 27 g magnesium), and one medium banana (31 g magnesium) were significantly correlated with male participants.

The study observed that as the BMI increased, there was a notable increase in the consumption of certain magnesium-rich foods. This included one slice of brown bread containing 24 g of magnesium in a 30 g serving and a cup of beans with 84 g of magnesium. Similarly, a cup of spinach (24 g magnesium), a cup of okra (74 g magnesium), and a can of tuna fish (20 g magnesium in 185 g) were more frequently consumed by individuals with higher BMI. Additionally, liver (15 mg magnesium in 30 g), an egg (14 g magnesium), half a cup of pumpkin seeds (84 g magnesium), a tablespoon of peanut butter (27 g magnesium), 8-10 grains of cashew nuts (178 g magnesium), a medium banana (31 g magnesium), and either one large or three small, dried figs (55 g magnesium) were also associated with increased BMI levels.

## DISCUSSION

Despite the detailed analysis of magnesium-containing food consumption in our study sample, a significant finding was the low dietary magnesium intake, with most participants consuming it only 1 to 5 times monthly. Low intake of magnesium is common worldwide. It has been observed that the typical diet in many industrialized countries, often referred to as a standard Western diet, frequently falls short of providing the recommended magnesium levels. For instance, a study in the United States reported that 67% of women and 64% of men do not meet the recommended levels of magnesium intake [19].

Our study primarily involved young adults, predominantly students, and this age category is highly exposed to unhealthy eating behavior [20]. This finding agrees with a previous Saudi report, which found lower magnesium consumption than the recommended daily needs among medical students at a university in Northwestern Saudi Arabia [21]. Research indicates that typical Saudi dietary patterns mainly include wheat bread, white rice, dates, and Arabic coffee [22]. Several factors contribute to the low dietary magnesium intake in Saudi Arabia. One significant factor is the limited availability and consumption of whole grains, which are a rich source of magnesium. Modern dietary preferences, shaped by urbanization and the influence of Western eating habits, have increased consumption of processed and fast foods, potentially leading to a decreased intake of magnesium-rich foods, legumes, vegetables, fruits, and dairy products [23]. Thus, whole grain products, such as brown rice, whole wheat bread, and oatmeal, are commonly replaced with refined grains, which undergo a milling process that removes the bran and germ, reducing magnesium content.

Another factor is the preference for meat-based diets, which can contribute to inadequate magnesium intake. While meat can provide certain nutrients, it is relatively low in magnesium compared to plant-based sources such as legumes, nuts, and seeds. Carbonated beverages, popular in Saudi Arabia [24], can also displace magnesium-rich beverages like water, further contributing to low magnesium intake. Furthermore, a local study evaluated the concentration of six magnesium imported brands, and eight sources of natural drinking water along with 12 local bottled water brands and found that the local bottled water had suboptimal magnesium levels [25]. The general population in the Eastern Province depends mainly on desalinated drinking water, which could be another lifestyle factor that affects magnesium status [9].

In our study, magnesium intake was insufficient in a significant portion of the sample, particularly among specific demographic groups such as males, younger individuals, and those with a higher BMI. This trend can be attributed to the recent lifestyle changes across Saudi Arabia, especially among the younger generation [21]. In terms of the lifestyle or socioeconomic features associated with hypomagnesemia, several factors have been documented in the literature, such as gender, age, smoking, and physical activity. Previous studies showed controversies regarding the association between hypomagnesemia and gender. For instance, Levy *et al*. reported higher magnesium intake among women compared to male participants [26]. Two other studies concluded that female participants consumed lower daily magnesium than male participants [27,28]. Thereby, the association between hypomagnesemia and gender is inconclusive. In this study, we also investigated gender-specific patterns in magnesium intake. We found that the consumption of certain dietary sources of magnesium was more commonly associated with men, while other sources were predominantly consumed by women. The consumption of oats, quinoa, lentils, spinach, chia seeds, and pecans was significantly correlated with women. In contrast, brown rice, chickpeas, beans, black beans, liver, peanut butter, and banana were significantly correlated with men. Concerning the relationship between hypomagnesemia and age, Levy *et al*. [26] documented a higher dietary magnesium intake among older participants (>60 years). Conversely, Ford *et al*. [27] indicated that the median magnesium daily intake was lower than the estimated average requirement and recommended dietary allowance for all age groups except Caucasian men between 31 and 50. Conversely, this study concluded that daily dietary magnesium intake increased with age.

An inverse relationship between obesity and magnesium intake was documented by Levy *et al*. [26] who showed that overweight/obese patients were more likely to consume less magnesium compared to individuals who had normal weight. However, the study highlighted that although there was a relationship between low dietary magnesium intake and obesity, it remains uncertain whether this finding reflects poor diet among obese participants or whether hypomagnesemia is a risk factor for obesity [26]. In a different approach, Dominguez *et al*. [29] concluded that obesity increases the risk of hypertension in patients with low magnesium intake compared to individuals with normal weight. This study found that patients with increased BMI were more likely to consume certain types of food containing magnesium, including brown bread, beans, spinach, okara, tuna fish, liver, egg, pumpkin seeds, peanut butter, cashew nuts, bananas, and dried figs.

Evidence suggests that magnesium consumption in the Eastern Province of Saudi Arabia falls below the levels recommended by the Institute of Medicine [30]. The prevalence of inadequate dietary magnesium consumption appears to be a global problem. For instance, studies have shown that 82% and 99.5% of residents in Sao Paulo [28] and 56%-68% in the United States have inadequate magnesium intake [27]. The same profile appears in most African countries [31]. Given the numerous biological benefits of magnesium, increasing its intake through dietary supplements could serve as a significant source of this essential mineral. Current nutritional recommendations encourage the adequate intake of fruits and vegetables and emphasize the benefits of nut consumption since inadequate magnesium is linked with an increased risk of several health issues.

This study has several limitations that should be considered when evaluating the results. Although this study used an online survey for data collection, it may introduce selection bias. Another significant limitation is the age distribution of the participants. Most were younger adults aged 39 years or below, largely due to the recruitment methods being concentrated on a university campus. Further research is needed to evaluate the magnesium intake levels in younger (<18) and older (>65) ages who were not examined in the present study.

## CONCLUSION

The research highlights the prevalence of low dietary magnesium intake in the Eastern Province of Saudi Arabia. Addressing this issue is crucial to improving the overall health and well-being of the population. Therefore, it is important to implement regular surveys to monitor magnesium levels among residents that could potentially contribute to the early detection of subclinical hypomagnesemia and its related health risks. Educational programs for the general public and/or health care providers regarding the physiological importance of magnesium, diagnosis of subclinical magnesium deficiency, and promotion of magnesium-rich foods consumption can help raise awareness. Further research using a larger cohort is needed to assess the current magnesium intake levels in different age groups and regions of Saudi Arabia and to develop targeted interventions for improving magnesium consumption.

## Data Availability

Data is available from the corresponding author upon request.
